# Personal Responsibility in Contagious Diseases

**Published:** 1886-06

**Authors:** 


### PERSONAL RESPONSIBILITY IN CONTAGIOUS DISEASES.

Judge Dixon, of New Jersey, in a recent charge to the grand jury at Paterson, said : "If a man, conscious that he carries about with him the germs of contagious disease, recklessly exposes the health and lives of others, he is a public nuisance and a criminal, and may be held answerable for his conduct. If death occurs through his recklessness he may be indicted for manslaughter. It is held, that, where a person knowingly communicates a contagious disease to another, and death results, the crime is manslaughter." Judge Dixon added : "The man may be indicted also for spreading the disease by conscious exposure of others thereto by his presence in public places, such as on the streets, in halls, etc. He might be indicted as a public nuisance for endangering the public health in this way, even if no consequence had followed. The law provides some penalty for such offenses against the public safety."

"While for obvious reasons," says Bishop in his *Criminal Law*, "a man is not punishable for being sick of a contagious disease in his own house, though the house stands in a popular locality, and while his friends are not guilty of crime in declining to remove him, yet if the sick man goes out into the public way, carrying with him his infection to the danger of the public, or if one takes out an infected child, this act, at the common law, subjects the doer to an indictment."

The house infected with a contagious disease should be marked in such a way as to warn the public ; and, after the disease has disappeared, public safety demands that disinfection should be thoroughly and intelligently done. To accomplish this local sanitary associations are an absolute necessity. Mr. Simon, the English sanitarian, says that the following principles should be embodied in the common law :—

"1. That each case of such disease is a public danger, against which the public, as represented by its local authorities, is entitled to be warned by proper information. 2. That every man who, in his own person, or in that of any one under his charge, is the subject of such disease, or is in control of circumstances relating to it, is, in common duty toward his neighbors, bound to take every care which he can against the spreading of the infection ; that, so far as he would not of his own accord do his duty, his neighbors ought to have ample and ready means of compelling him ; and that he should be responsible for giving to the local sanitary authority notification of his case, in order that the authority may, as far as needful, satisfy itself as to the sufficiency of his precautions. 3. That, so far as he may, from ignorance, not understand the scope of his precautionary duties, or may, from poverty or other circumstances, be unable to fulfill them, the common interest is, to give him liberally out of common stock such guidance and such effactual help as may be wanting. 4. That, so far as he is voluntarily in default of his duty, he should not only be punished by penalty as for an act of nuisance, but should be liable to pay pecuniary damages for whatever harm he occasions to others. 5. That the various undertakings which in certain contingencies may be instrumental in the spreading of infection, water companies, dairies, laundries, boarding-schools, lodging-houses, inns, etc., should respectively be subject to special rule and visitation in regard to the special dangers they may occasion, and that the persons in authority in them should be held to strict account for whatever injury may be caused through neglect of rule. 6. Finally, that every local sanitary authority should always have at command, for the use of its district, such hospital accommodation for the sick, such means for their conveyance, such mortuary, such disinfection, establishment, and, generally, such planned arrangements and skilled service, as may, in case of need, suffice for all public requirements of the district."—*Science News.*

